# Equitable pathways: Reimagining behavioral health care transitions applying a transition of care bundle model

**DOI:** 10.1097/nmg.0000000000000369

**Published:** 2026-05-29

**Authors:** Jenny Bernard, Jazmin Cascante, Aimee Gabuya, Victor Carrillo

**Affiliations:** At the Hackensack Meridian Health Network Department of Patient Safety & Quality in Hackensack, NJ, **Jenny Bernard** is VP, Women and Community Health Quality; **Jazmin Cascante** is Director of Patient Safety & Quality - Women's Service; **Aimee Gabuya** is Research Nurse Coordinator of the First Thirty quality improvement project; and **Victor Carrillo** is Senior Vice President & Associate Chief Quality Officer. Dr. Bernard and Dr. Carillo are also affiliated with the Hackensack Meridian School of Medicine in Nutley, NJ.

**Keywords:** care coordination, discharge planning, hospital readmission, social drivers of health (SDoH), substance use disorder (SUD), transition of care (TOC)

## Abstract

**Background:**

Transitions from inpatient to outpatient care pose significant risks for individuals with behavioral health conditions (BHC), contributing to fragmented care and preventable hospital readmissions. Comprehensive transition of care (TOC) interventions that address both clinical and social needs may improve outcomes in this vulnerable population.

**Purpose:**

To evaluate the effectiveness of the First Thirty (FT) program, a nurse-led TOC bundle, in reducing hospital readmissions among patients with BHC and/or substance use disorder (SUD).

**Methods:**

This retrospective chart review included adults aged 18 to 64 years with Medicaid or no insurance enrolled in the FT program across 10 hospitals from 2022 to 2023. The FT program applied a TOC bundle comprising care coordination, medication support, discharge phone calls, transportation assistance, and wellness resources. Hospitalizations during the 30 days before and after program enrollment were compared, with analyses conducted separately for 2022 and 2023 cohorts.

**Results:**

Among 3079 patients, readmission rates declined significantly after FT enrollment in both cohorts. In 2022, readmissions decreased from 12.7% pre-enrollment to 6.9% post-enrollment, and in 2023 from 8.2% to 3.3% (both *P* < .0001), despite differences in baseline cohort characteristics.

**Conclusions:**

The FT TOC model was associated with substantial reductions in 30-day readmission, suggesting that structured, nurse-led care coordination can improve transitions and outcomes for patients with BHC and SUD.

**Figure:**
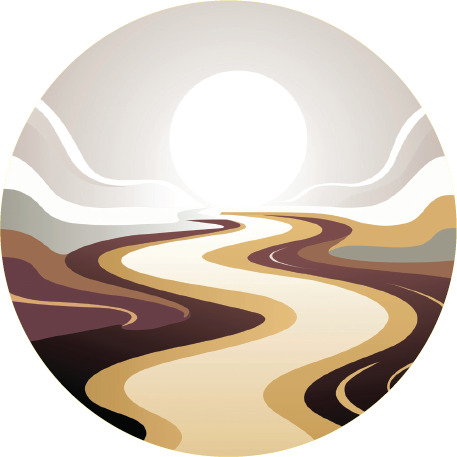
No caption available.

Mental illness affects millions of Americans, with an estimated one in five adults experiencing some form of mental illness annually.^1^ In the US, it's estimated that close to 60 million adults live with mental illness.^1^,^2^ Additionally, more than 40 million Americans suffer from substance use disorder (SUD), with more than 100 000 deaths attributable to overdoses each year.^3^ Unfortunately, only 6.5% of those with SUD receive treatment, and rates of fatal overdose are highest among Black and Native American/Alaska Native populations, signaling a disparity in health outcomes.^3^ The prevalence of behavioral health conditions (BHC), which includes mental health, SUD, and other conditions, has also increased in recent years, reflected in threefold and fourfold increases in self-reported anxiety and depression, respectively.^4^

The treatment of BHC has shifted from extensive psychiatric inpatient hospitalizations to the utilization of outpatient and community services to alleviate the demand for psychiatric beds and decrease emergency care usage.^5^,^6^ For those discharged to the community after an inpatient stay for behavioral health treatment, the transition from a hospital to an outpatient setting poses significant risks.^7^ Individuals with behavioral health challenges visit the emergency department (ED) more frequently and face a higher risk of readmissions due to factors such as the severity of illness with cognitive impairment, difficulty with medication adherence, limited access to care, and lack of social support.^8^

Providing a smooth postdischarge process is crucial to ensuring the continuity of care, as individuals with BHC require comprehensive care management. Maintaining established relationships between the discharging hospital facility and the receiving outpatient services contributes to a successful transition by facilitating effective communication, coordinated care, and a smooth transfer of patient information and responsibility.^9^ Effective discharge planning should start early during the index hospitalization, focusing on collaborative planning, patient education, timely follow-ups, medication management, coordination of care, family involvement, and the use of technology to improve overall care continuity between inpatient and outpatient teams.^9^ Setting a clear timeline for outpatient care enables patients to begin treatment sooner, reducing the duration of illness and enhancing the likelihood of avoiding readmissions.^10^

As demonstrated by a program implemented in Ontario, Canada, targeted efforts to implement evidence-based strategies to improve BHC care transitions can positively impact patient outcomes. That program, the Transitional Discharge Model, bridges care gaps for vulnerable mental health patients and supports their transition from inpatient settings back into the community.^11^ This approach to care coordination, coupled with a valuable strategy, alleviates the problems of increased readmissions and ED visits, and was estimated to save the hospital almost $3.4 million.

Social factors can complicate care transitions for patients with BHC, underscoring the urgent need for a comprehensive transition of care (TOC) program that accounts for both clinical and social needs.^12^ Efforts to address social drivers of health (SDoH) when transitioning inpatients receiving treatment for mental health and SUD can reduce readmissions and improve aftercare.^13^

In an effort to improve outcomes for patients treated for BHC at the largest, multihospital health system in the state, a program was developed to provide comprehensive and collaborative care coordination that included a TOC Bundle (TOCB) to facilitate a seamless transition of patients with BHC from inpatient hospitalization to outpatient care treatment. The TOCB pulls from published evidence and prior learnings to incorporate specific elements intended to support patients, connect them to community services, and address their social and clinical needs.

The primary objective of this study was to assess the effectiveness of the program and the TOCB among patients discharged with BHC and SUD. This evaluation examined hospitalization frequency before and after program enrollment.

## METHODS

### First Thirty program

**FIGURE 1:**
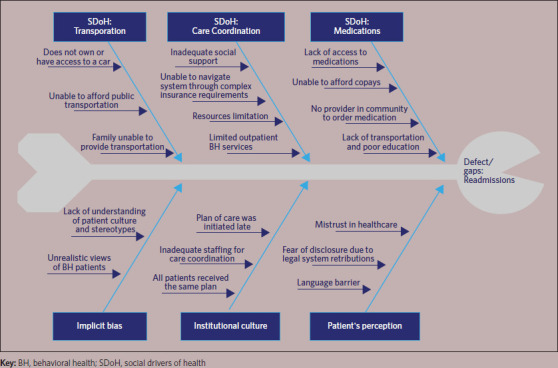
Fishbone diagram

**FIGURE 2:**
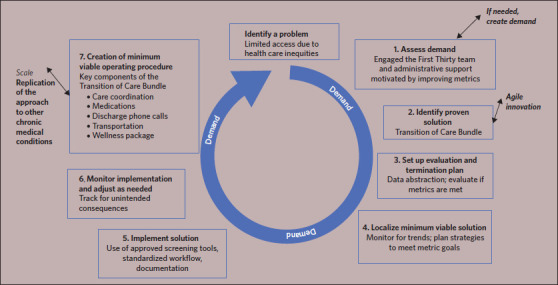
The Agile transformation process

A visionary nursing leader, recognizing the urgency of mitigating disparities in the care of BHC, took the initiative to establish and execute the First Thirty (FT) program, an innovative and intense care coordination program that involves monitoring patients for the “first 30” days after discharge. The program's development was guided by the Lean Six Sigma methodology, which included the use of a fishbone diagram to visualize and brainstorm ideas while exploring possible cause-and-effect relationships (see Figure 1).^14^ Additionally, Agile Science was employed in applying a collection of evidence-based interventions throughout this project (see Figure 2). Agile Science focuses on understanding, predicting, and influencing individual and organizational behavior within complex real-world contexts, while emphasizing the importance of speed, context, and timing in the flow of information and resources.^15^

During the creation of the FT program, the team developed a TOCB specifically designed for patients hospitalized with BHC. The nurse leader used a team approach to identify the main problem and the factors contributing to the problem. Through an in-depth analysis of critical theme issues and lessons from a successful TOCB developed during the COVID-19 pandemic, the team created a TOCB that incorporates five interventions grounded in a patient-centered care philosophy. These interventions include care coordination, medication support through an adherence program, discharge phone calls, transportation provisions for follow-up care, and an individual wellness package. The package includes items such as automatic and battery-powered self-monitoring devices, winter gloves and hats, and disposable warming blankets. Nursing staff were integral to the development and implementation of these interventions, by providing crucial information to coordinate care, making discharge phone calls, and ensuring transportation arrangements were adequate. Nurses were also primarily responsible for communicating directly with patients about these interventions.

With the TCOB in place, the FT team of nurses, care navigators, and transition assistants could identify patients hospitalized for BHC early in their stay who were eligible for enrollment in the FT program. Approval from the Institutional Review Board (IRB) was obtained prior to launching the FT program to ensure compliance with ethical standards.

During bedside interactions, nurses screened enrolled patients for alcohol and substance use, and SDoH needs were identified and documented for use during discharge planning. The development and delivery of care plans for enrolled patients incorporated input from community-based organizations (CBOs). In addition to building partnerships with CBOs, this input ensured that care delivery during and after discharge addressed patients' SDoH needs, using evidence-based guidance for navigating the various nonmedical conditions that affect health outcomes, including the environments in which individuals are born, grow, work, live, worship, and age.^16^

At discharge, referrals to the CBOs for care were strategically made across three regions of the health network—northern, central, and southern—based on the patient's geographic location, and transportation was arranged. Nursing staff, under the supervision of a nurse leader, primarily coordinated these referrals. Patients for whom specific SDoHs were identified as inadequate were provided with readily available resources, including food insecurity gift cards, a 30-day supply of medications through a Meds-to-Beds program shown to improve medication adherence, and transportation coordination for follow-up appointments and trips to community services such as food pantries.^17^

**FIGURE 3:**
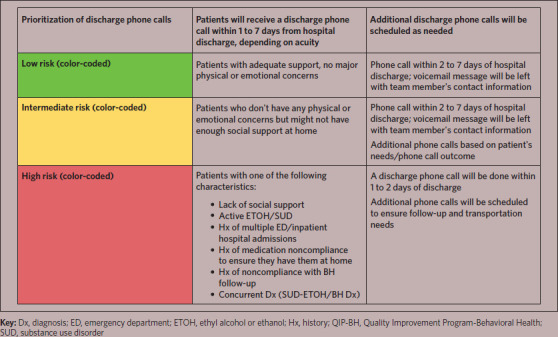
QIP-BH discharge phone call process

Postdischarge, an FT team member (usually a nurse) conducted regular follow-up phone calls with discharged patients. These conversations may have included appointment reminders, assistance with medication refills, and wellness checks. The timing and frequency of these phone calls depended on specific patient factors collected by the FT team during the patient's stay and on preestablished and clear criteria (determined jointly by FT leadership and the team) for prioritization (see Figure 3).

Patients with adequate support and no major physical or emotional concerns received a call within 2 to 7 days after discharge, and the FT team member also left their contact information so that the patient could reach them if needed. Those without any physical or emotional concerns, but who lacked sufficient social support at home, also received a call within 2 to 7 days, and additional phone calls were scheduled as needed based on patient needs and the substance of the initial call. Patients with an active alcohol or SUD, concurrent SUD/BHC diagnosis, a history of multiple ED or inpatient admissions, or a history of noncompliance with medications or BHC follow-up received a call within 1 to 2 days, with additional calls scheduled to support patient needs regarding transportation and follow-up appointments.

Regular follow-up calls were also made to community partners and CBOs to verify patient attendance at scheduled appointments. This ensured patient adherence to the postdischarge plan. Although the team was open to adjusting components of the program during operation based on initial results, no major structural changes to the program became necessary. However, some operational workflows were refined as the program grew to support efficiency and consistency across all sites.

### Study design and patient population

This study involved a retrospective chart review at 10 hospitals within a single health system, including academic and community hospital settings. Eligible patients were individuals aged 18 to 64 years with no insurance or Medicaid coverage and with BHC and/or SUD enrolled in the FT program between January 1, 2022, and December 31, 2023. The program started at only a few hospitals and gradually expanded as new team members were onboarded. This resulted in increased patient enrollment and volume over the study period.

### Measures and outcomes

Information obtained from each patient included clinical diagnoses, payor sources, services provided, and length of stay (LOS). Also collected were hospital admissions during the 30 days prior to and following enrollment in the FT program. The 30-day period was chosen because it reflects a common metric in quality measure programs. Additionally, program components were aligned with state metrics. Patient demographics, including race, ethnicity, gender, zip code, and county, were abstracted from the electronic health record. Only trained and designated chart abstractors collected these data, and abstractors utilized a double data entry methodology to minimize errors in abstraction.

### Statistical analysis

Descriptive statistics for continuous variables include mean and standard deviation when data are normally distributed and median and interquartile range (IQR) otherwise. Counts and percentages are used to describe categorical variables. The assumption of normality of continuous variables was assessed using the Shapiro-Wilk test of normality when the sample size for a characteristic was less than 2000 and the Kolmogorov-Smirnov test when sample sizes were larger than 2000. Comparisons of continuous variables were done with either a two-sided two-sample *t*-test (for normally distributed data) or a two-sided Wilcoxon rank-sum test (for nonnormal data). Comparisons of the percentages of patients with ED visits and hospitalizations before and after FT program enrollment were tested using McNemar's Test, which is appropriate for paired nominal data. Because the FT program was implemented over a period of time during 2022 and not fully implemented until 2023, analyses were performed separately for patients enrolled in 2022 and those enrolled in 2023. Statistical analyses were performed using SAS version 9.4 (SAS Institute Inc., Cary, NC, US).

## RESULTS

This analysis included 3079 patients, with 1330 patients from 2022 and 1749 from 2023. The median age was 38 (IQR: 29-50) years in 2022 and 40 (IQR: 31-51) years in 2023 (*P* = .0008, see Table 1). The distribution of gender was similar across the 2 cohort years, whereas race and Hispanic ethnicity differed: 2023 had a lower percentage of Black patients and a higher percentage of Hispanic patients. The 2023 cohort year also had fewer Medicaid patients (62.04% vs 66.77%) and more self-pay patients (22.47% vs 16.92%, *P* = .0010). Characteristics of the index stay were similar across cohort years: median LOS was 6.0 days, and approximately 89% were discharged home. The percent of patients with an alcohol use disorder and SUD was similar in 2022 and 2023, but the 2023 cohort had fewer patients with serious mental illness (82.73% vs 86.39%, *P* = .0057, see Table 1).

**TABLE 1:** Patient characteristics

Characteristic	2022 Cohort (n = 1330)	2023 Cohort (n = 1749)	*P* value
Age, years			.0008
Median (IQR)	38.0 (29.0-50.0)	40.0 (31.0-51.0)	
Gender, n (%)			.6061
Female	520 (39.10)	715 (40.88)	
Male	800 (60.15)	1021 (58.38)	
Transgender	10 (0.75)	13 (0.74)	
Race, n (%)			.0002
Native American /Alaska Native	0 (0.00)	5 (0.29)	
Black or African American	266 (20.00)	320 (18.30)	
Asian	33 (2.48)	62 (3.54)	
Caucasian	634 (47.67)	831 (47.51)	
Other	385 (28.95)	479 (27.39)	
Unable to obtain	12 (0.90)	52 (2.97)	
Ethnicity, n (%)			.0010
Not Spanish/Hispanic/Latino	1001 (75.38)	1214 (69.41)	
Spanish/Hispanic/Latino	317 (23.87)	514 (29.39)	
Declined/Unable to obtain	10 (0.75)	21 (1.20)	
Insurance class, n (%)			.0017
Self-pay + no insurance	225 (16.92)	393 (22.47)	
Medicaid	888 (66.77)	1085 (62.04)	
Dual eligible^a^	72 (5.41)	99 (5.66)	
Unknown	145 (10.90)	172 (9.83)	
Length of stay, days			.1350
Median (IQR)	6.0 (4.0,9.0)	6.0 (4.0,9.0)	
Discharged to home, n (%)			.5391
Not discharged to home	142 (10.68)	199 (11.38)	
Discharged to home	1188 (89.32)	1550 (88.62)	
ETOH use disorder, n (%)	449 (33.76)	640 (36.59)	.1034
Substance use disorder, n (%)	469 (35.26)	586 (33.50)	.3085
Serious mental illness, n (%)	1149 (86.39)	1447 (82.73)	.0057

a Eligible for both Medicare and Medicaid

**Key:** BHP, behavioral health provider; ETOH, ethyl alcohol or ethanol; IQR, interquartile range; SD, standard deviation

**FIGURE 4:**
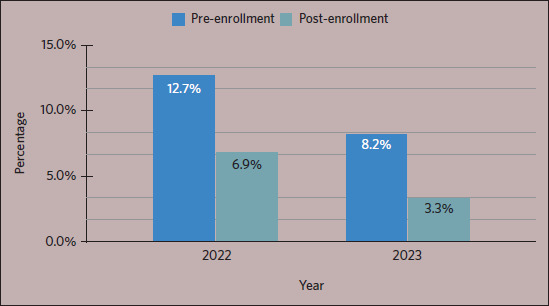
Behavioral health 30-day readmissions—inpatient (2022 and 2023)

The FT program was associated with a significant reduction in hospitalizations in both 2022 and 2023, cutting the percentage of patients with an inpatient hospital visit by approximately half from before to after program enrollment (see Figure 4). For patients enrolled in 2022, 12.7% experienced a hospitalization in the 30 days prior to enrollment in the FT program compared with 6.9% thereafter (*P* < .0001). For those enrolled in 2023, those percentages were 8.2% and 3.3%, respectively (*P* < .0001). The difference in the pre-enrollment hospitalization rate between 2022 and 2023 (12.7% vs 8.2%) was statistically significant (*P* < .0001).

## DISCUSSION

The transition from inpatient care for BHC to the community setting poses significant challenges for patients, often resulting in undertreatment following discharge. Studies have reported that only about half of patients discharged from inpatient psychiatric treatment attend a mental health outpatient visit within 7 days, and that communication between discharging providers and outpatient providers is often lacking.^18^ Medication adherence and access to outpatient treatment can significantly reduce relapse and readmission.^9^ Receiving timely follow-up from physicians can greatly reduce the risk of psychiatric readmission, and higher care continuity during the transition process is associated with better patient outcomes.^19^-^21^

Focusing care on immediate and specific patient needs alleviates the uncertainty about what may happen after leaving the hospital.^22^ Additionally, individuals with BHC and their families have cited the importance of considering human and social factors in the discharge planning process, including the value of social networks and support during the transition period.^7^ The role of nursing staff was critical in the success of this program, given the frequency with which nurses interact with patients and their ability to organize and coordinate the necessary components of the intervention.

The findings of the current study confirm that the FT program, which incorporates evidence-based processes and tools for easing patient transition described in the literature, is effective. In 2022 and 2023, the FT program was associated with significantly fewer hospitalizations within the 30 days after program enrollment compared with 30 days prior to program enrollment. This finding aligns with previous studies in which targeted efforts to improve this care transition reduced readmissions and ED visits, as well as costs.^11^,^21^ The TOCB used in the FT program specifically addresses the key challenges and barriers to transitioning individuals with BHC from the hospital to the community: care coordination (to ensure continuity), medication adherence, clinician follow-up, and addressing social and human factors, including SDoH.

It isn't immediately clear why the pre-enrollment hospitalization rate was lower in 2023 than in 2022, given that the two cohorts were of similar age, with similar percentages of alcohol use disorders and SUD. Additionally, the median LOS of the index hospitalization was the same, and a similar percentage of patients were discharged home. However, the 2023 cohort had a lower percentage of patients with serious mental illness and fewer with Medicaid coverage, which could have played a role in the lower pre-enrollment hospitalization rate compared with the 2022 cohort.

Regardless, both the 2022 and 2023 cohorts demonstrated a significant reduction in hospitalization rates from pre-enrollment to post-enrollment, suggesting that the program's impact is robust despite differences in cohort characteristics and pre-enrollment hospitalization rates. In general, the results of this 2-year study clearly demonstrate that the delivery of the FT TOC model is effective in addressing the needs of patients with Medicaid or no insurance transitioning from inpatient care for BHC to the community setting.

### Strengths and limitations of the study

Strengths of this study include the use of a large sample of individuals with BHC over a 2-year period and across 10 different hospitals. Another strength is the use of manual chart review to identify health encounters before and after FT enrollment. A limitation is that the present study includes only patients with Medicaid or no insurance and may not be generalizable to those with other types of insurance.

## IMPLICATIONS FOR NURSE LEADERS

The scientific underpinning of this care coordination reflects the evidence for an innovative approach to the care continuum. Nurse leaders play a pivotal role in addressing SDoH and uplifting the voice of nursing in the marginalized population nurses serve, and therefore leaders need to leverage hospital and community resources with targeted patient needs. Behavioral health care is complex, and it's essential for nurse leaders to continually evaluate the process against its intended outcomes and adapt care to achieve the desired goals. Nurse leaders are well-positioned to promote patient safety and excellence in care that corresponds to the organization's strategic plans. Given its success, we recommend a strategic scaling plan for this novel TOC model by piloting its replication under other conditions.

## EFFECTIVE AND ATTAINABLE SOLUTIONS

The FT program integrates trailblazing solutions that impact patient care and are realistic and achievable for patients diagnosed with BHC, including SUD. The program primarily leverages existing resources and expands linkages with community service providers to reduce hospitalizations. The FT program empowers nurse leaders to create a sustainable change in the care coordination continuum while ensuring patient autonomy, strengthening care relationships, and facilitating access to resources. The innovative care model employs a holistic approach, effectively reducing hospital readmissions in a vulnerable population.

## Equitable pathways: Reimagining behavioral health care transitions applying a transition of care bundle model

### TEST INSTRUCTIONS

- Read the article. The test for this nursing continuing professional development (NCPD) activity is to be taken online at www.Nursing Center.com/CE
- You'll need to create an account (it's free!) and log in to access My Planner before taking online tests. Your planner will keep track of all your Lippincott Professional Development online NCPD activities for you.
- There's only one correct answer for each question. A passing score for this test is 8 correct answers. If you pass, you can print your certificate of earned contact hours and access the answer key. If you fail, you have the option of taking the test again at no additional cost.
- For questions, contact Lippincott Professional Development: 1-800-787-8985.
- Registration deadline is **June 2, 2028**.

### PROVIDER ACCREDITATION

Lippincott Professional Development will award 2.0 contact hours for this nursing continuing professional development activity.

Lippincott Professional Development is accredited as a provider of nursing continuing professional development by the American Nurses Credentialing Center's Commission on Accreditation.

This activity is also provider approved by the California Board of Registered Nursing, Provider Number CEP 11749 for 2.0 contact hours. Lippincott Professional Development is also an approved provider of continuing nursing education by the District of Columbia, Georgia, West Virginia, New Mexico, South Carolina, and Florida, CE Broker #50-1223. Your certificate is valid in all states.

**Payment:** The registration fee for this test is $21.95.
